# Appendix Bleeding with Painless Bloody Diarrhea: A Case Report and Literature Review

**DOI:** 10.1515/med-2019-0084

**Published:** 2019-10-02

**Authors:** Wanqun Chen, Hong Qiu, Xiaojun Yang, Jinwei Zhang

**Affiliations:** 1 Department of dermatology and cosmetology, Chongqing Hospital of Traditional Chinese Medicine, Chongqing 400037, China; 2 Department of Gastroenterology, Chongqing Hospital of Traditional Chinese Medicine, Chongqing 400037, China

**Keywords:** Appendix bleeding, Granulomatous appendicitis, Painless bloody diarrhea

## Abstract

Appendix bleeding is an uncommon clinical phenomenon. In this article, we reported a case of appendix bleeding with painless bloody diarrhea. With the analysis of clinical features, clinical examination, experimental test and literature review, we diagnosed that the appendix bleeding might be caused by granulomatous appendicitis. This successfully cured case might be a reference for later diagnosis and treatment of appendix bleeding with painless bloody diarrhea.

## 1 Introduction

It’s well known that lower gastrointestinal (GI) bleeding generally refers to bleeding from the colon and anorectum, also including bleeding from the small intestine (from the level of major duodenal papilla till the distal ileum). In most patients, the lower GI bleeding stops spontaneously and does not recur, but some cases of lower GI bleeding may be life-threatening [1]. Therefore, it’s worthy to investigate the rare case of lower GI bleeding to guide for clinical practice. In this study, we reported a case of appendix bleeding with painless bloody diarrhea. Through the analysis of clinical features, contrast-enhanced CT findings, angiography findings, colonoscopy findings, surgical outcomes, pathological tissue, patient follow-up and literature review, we believed that granulomatous appendicitis may be the cause of appendix bleeding.

## 2 Case report

A 24-year-old male patient came to our department for a 5-hour dark red stool. On admission, the patient had an acute ill looking a ppearance without mental disorder or resting tachycardia. The results of blood pressure test and chest examination were normal. The abdomen was soft with normal bowel sounds. The abdomen can be touched without tenderness and there was no organomegaly in abdomen. The patient had neither fever nor abdominal pain and he denied any history of gastrointestinal diseases or medicine taking. But reddish bloody stools occurred once in 3 hours, approximately 100-200 ml of blood each time.

On admission, laboratory evaluation revealed a white blood cell count of 6.16*109/L, hemoglobin of 138g/L, and platelet count of 111*109/L. Prothrombin time was 12.5 seconds with international normalized ratio of 1.08, activated partial thromboplastin time was 28.8 seconds. The liver and renal function, electrolyte were normal, and C-Reactive Protein was 2.9mg/L. 8 hours after admission, the hemoglobin level dropped from 138g/L to 90 g/L，so emergency gastroscope was administered. However, the result of gastroscope showed no specific lesion in esophagus, stomach and duodenum.

**Figure 1:**
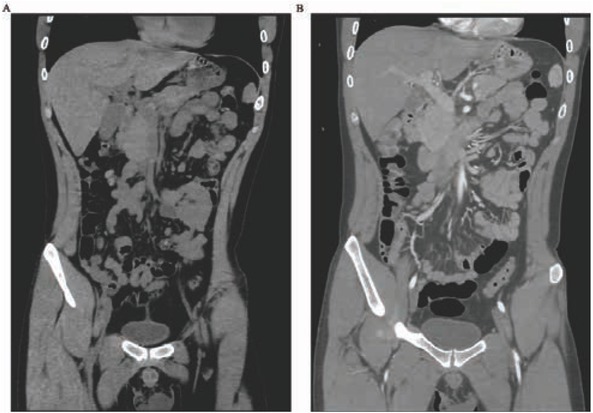
No lesion was found in the Contrast-enhanced CT and the appendix displayed normal.

24 hours after admission, although intravenous proton pump inhibitors (Esomeprazole, 80mg bolus followed by 8mg/hour); somatostatin (0.25mg bolus followed by 0.25mg/hour) and thrombin three or four times/day; additionally with vitamin, glucose and potassium chloride were administered, the frequency of rectal bleeding increased to once in 2 hours. So contrast-enhanced CT and angiography were performed on the abdomen and superior mesenteric artery to find the source of bleeding. But no lesion was found (Fig. 1).

**Figure 2:**
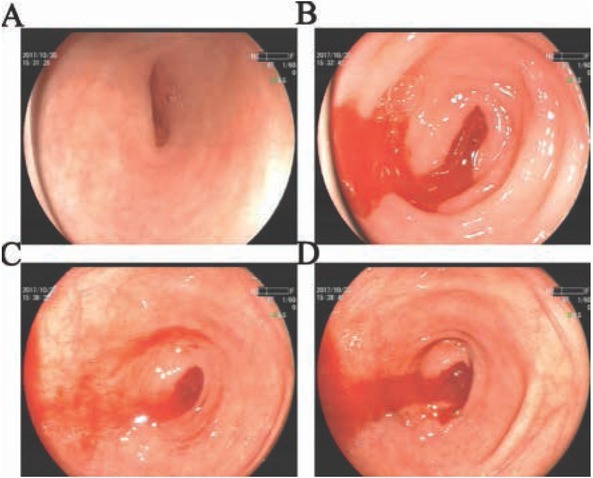
Colonoscopy showed that blood came from the appendiceal orifice with time. (A) Irrigation washed away the blood. (B) Oozing bright blood puffed at the appendiceal orifice. (C) Repetitive irrigation couldn’t clear away the blood. (D) After about 10 seconds, oozing bright blood flowed away from the appendiceal orifice.

In order to find the cause of bleeding, colonoscopy was subsequently performed. We saw a large amount of fresh blood filling in the enteric cavity even in the terminal

ileum through colonoscopy. When the colonoscope got back from ileum to ileocecal junction, we found the color of blood got fresher. So irrigation was performed within the ileocecal junction. After the blood was washed away, oozing bright blood flowed from the appendiceal orifice immediately (Fig. 2).

On the third day from admission, an emergency appendectomy was arranged. On operation, a normal appearance of appendix filled with blood was revealed, but no edema or abscess. In the operation, a colonoscopy with repetitive irrigation was performed. A large amount of blood clot and edema mucosa was revealed, but absent of longitudinal pattern of ulceration or cobble-stone appearance of mucosa.

After appendectomy, the patient was not experiencing bloody stools or other discomfort. Then discharged.

**Figure 3:**
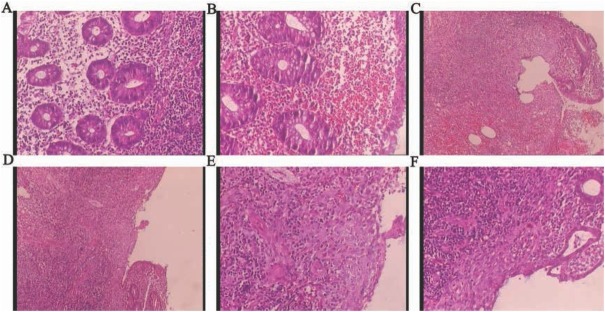
The histology of the appendix after surgery. (A) Inflammatory cells infiltrating. (B) A large number of red blood cells could be seen. (C) Ulcer, inflammatory cells and granulomas were visible. (D-F) Scattered noncaseating granulomas could be seen.

The HE staining of the appendix found a number of inflammatory cells and non-caseating granulomas (Fig. 3).

One year after the appendectomy, a follow-up survey was made. The patient denied abdominal pain, diarrhea and any discomfort.

**Ethics approval and consent to participate**: Patient provided informed consent and this report was approved by the Chongqing Hospital of Traditional Chinese Medicine Ethics Committee.

## 3 Discussion

By searching the PubMed/MEDLINE database, the relevant publications from January 1977 to May 2018 were identified by using the following searching term “appendix bleeding” or “appendix hemorrhage”. All the pub

lication included were in English, while those in other language were excluded. By searching the database, 17 articles were included as the following(Table 1).

**Table 1:** The information of the 17 articles published about appendix bleeding.

Year	Age/gender	C/C	Clinical impression	Ref.
2017	46/M	Abdominal pain	Appendicitis	[8]
2017	33/M	abdominal pain	Diverticulitis	[9]
2016	72/M	hematochezia	Angiodysplasia	[10]
2016	22/M	rectal bleeding	Granulomatous appendicitis	[11]
2015	68/M	hematochezia	Appendiceal Dieulafoy lesion	[12]
2014	44/M	hemorrhage	Diverticulitis	[7]
2014	51/M	chief complaint	Dieulafoy lesion	[13]
2013	71/M	melena	Appendix ulcer	[14]
2013	41/M	melena	Atypical florid vascular proliferations	[15]
2012	59/F	rectal bleeding	Aortoenteric fistula	[16]
2011	25/M	hematochezia	Focal erosion of appendix mucosa	[17]
2010	42/M	hematochezia	Appendiceal mucosal erosion	[18]
2007	56/M	hematochezia	Gastrointestinal stromal tumor	[19]
2001	76/F	bleeding	Angiodysplasia	[20]
1985	32/F	rectal bleeding	Ulcerated appendiceal stump	[21]
1980	48/M	bleeding	Diverticular hemorrhage	[22]
1977	14/M	bloody stool	Appendix abscess	[23]

M: male, F: female, C/C: chief complaint, Ref: reference

From the table, we could see the factors of appendix bleeding included inflammation, angiodysplasia, diverticulum，granulomatous appendicitis, tumor and damage of the appendix mucosa which were according with the previous description [2, 3]. Fifteen of these reports were of painless bloody diarrhea.

In our case report, the young patient presented with bloody diarrhea without any other positive symptom or examination result, except his hemoglobin declined from 138g/L to 90 g/L. From the data above, normal gastrointestinal endothelium was revealed by gastroscope and colonoscope, also with no vascular anomaly found by selective angiography, then angiodysplasia was ruled out. Besides, the normal number of platelet count, coagulation and fibrinolysis excluded the possibility of spontaneous hemorrhage result from thrombocytopenia, disseminated intravascular and defibrination[4]. Furthermore, negative result of contrast-enhanced CT scan excluded the diagnosis of appendicitis or tumor (Fig. 1 and 2). As a result, the cause of appendix mucosa damage and granulomatous appendicitis were under consideration. While the morphology and histology of appendix after surgery gave some clues for us. The characteristics of chronic inflammatory lesions including clusters of epithelioid histiocyte accompanied by multinucleated giant cells and lymphocytes as well as plasmatic cells were the hint of granulomas (Fig. 3).

As far as we know, this was the second case which revealed painless bloody diarrhea because of granulomatous appendicitis. Granulomatous appendicitis was a rare case of disease manifested with inflammatory lesion caused by fungi infection, yersinia pseudotuberculosis, mycobacterium tuberculosis, parasites, Crohn’s disease (CD), foreign body reactions, and sarcoidosis [3]. The exact cause of this case was obscure because it was used to be reported as a manifestation of CD [5, 6]. However the diagnosis of CD was very difficult even if several months after the start of the symptoms, not to mention the characteristics of CD in its early phase and its long-term natural history [5, 6].

Recently, it has been believed that granulomatous appendicitis was the subacute appendicitis managed conservatively [7]. In this study appendectomy successfully cured the bleeding and the patient was recovery based on a follow-up survey which showed no abdominal pain, no diarrhea or any discomfort. It was a successful case to make the diagnosis and differentiate diagnosis based on colonoscopy, contrast-enhanced CT scan and selective angiography.

## 4 Conclusion

In summary, we presented a case of lower GI bleeding caused by granulomatous appendicitis based on the analysis of clinical features, contrast-enhanced CT findings, angiography findings, colonoscopy findings, surgical outcomes, pathological tissue, patient follow-up and literature review. It suggested us that although granulomatous appendicitis was rare, it should be under consideration for the cause of lower GI bleeding. This successfully cured case might be a reference for later diagnosis and treatment of appendix bleeding with painless bloody diarrhea.

## References

[1] Ghassemi KA, Jensen DM (2013). Lower GI bleeding: epidemiology and management. Curr Gastroenterol Rep.

[2] Holtz LR, Neill MA, Tarr PI (2009). Acute Bloody Diarrhea: A Medical Emergency for Patients of All Ages. Gastroenterology.

[3] AbdullGaffar B. (2010). Granulomatous diseases and granulomas of the appendix. Int J Surg Pathol.

[4] Colovic M, Jurisic V, Colovic N, Miljic P (2003). Thrombotic thrombo-cytopenic purpura: clinico-pathologic characteristics and therapy. Srp Arh Celok Lek.

[5] Bischoff A, Gupta A, D’Mello S, Mezoff A, Podberesky D, Barnett S (2010). Crohn’s disease limited to the appendix: a case report in a pediatric patient. Pediatr Surg Int.

[6] Han H, Kim H, Rehman A, Jang SM, Paik SS (2014). Appendiceal Crohn’s disease clinically presenting as acute appendicitis. World J Clin Cases.

[7] Vesa TS, Hosseini-Carroll P, Manas K (2014). Diverticular hemorrhage of the appendix. Gastroenterol Hepatol (N Y).

[8] Shen Z, Huang YZ, Ning LM, Gao HC, Wang W (2017). A case of lower digestive tract hemorrhage caused by appendicitis in China. Int J Surg Case Rep.

[9] Ur Rehman M, Paulus F, Chew MH (2017). Unexpected histopathology of acute appendicitis. Int J Surg Case Rep.

[10] Choi JM, Lee SH, Lee SH, Ahn BK, Baek SU (2016). Hematochezia due to Angiodysplasia of the Appendix. Ann Coloprocto.

[11] Magaz Martinez M, Martin Lopez J, Dela Revilla Negro J, Gonzalez Partida I, de Las Heras T, Sanchez Yuste MR (2016). Appendicular bleeding: an excepcional cause of lower hemorrhage. Rev Esp Enferm Dig.

[12] Reynolds JK, Mejia VA (2015). Appendiceal Dieulafoy lesion: an unusual cause of massive lower gastrointestinal bleeding. Am Surg.

[13] Johnson A, Oger M, Capovilla M (2014). Dieulafoy lesion of the appendix. Dig Liver Dis.

[14] Konno Y, Fujiya M, Tanaka K, Sakatani A, Shimoda M, Hayashi A (2013). A therapeutic barium enema is a practical option to control bleeding from the appendix. BMC Gastroenterol.

[15] Gu MJ, Choi JH, Kim SH (2013). Atypical florid vascular proliferation in appendix: a diagnostic dilemma. Diagn Pathol.

[16] Monjero-Ares I, Gegundez-Gomez C, Rielo-Arias FJ, Alvarez-Gutierrez AE, Pena-Holguin J, Alvite-Canosa M (2012). Iliac-appendiceal fistula. An unusual cause of gastrointestinal bleeding. Rev Esp Enferm Dig.

[17] Chiang CC, Tu CW, Liao CS, Shieh MC, Sung TC (2011). Appendiceal hemorrhage -- an uncommon cause of lower gastrointestinal bleeding. J Chin Med Assoc.

[18] Baek SK, Kim YH, Kim SP (2010). Acute Lower Gastrointestinal Bleeding Due to Appendiceal Mucosal Erosion. Surg Laparosc Endosc Percutan Tech.

[19] Kim KJ, Moon W, Park MI, Park SJ, Lee SH, Chun BK (2007). Gastrointestinal stromal tumor of appendix incidentally diagnosed by appendiceal hemorrhage. World J Gastroenterol.

[20] Kyokane T, Akita Y, Katayama M, Kitagawa Y, Sato T, Shichino S (2001). Angiodysplasia of the appendix. Am J Gastroenterol.

[21] Choi DY, Yuh JN, Reid JD (1985). Rectal hemorrhage from ulcerated appendiceal stump nine years after appendectomy. Report of a case. Dis Colon Rectum.

[22] Norman DA, Morrison EB, Meyers WM (1980). Massive gastrointestinal hemorrhage from a diverticulum of the appendix. Dig Dis Sci.

[23] Milewski PJ (1977). Appendix abscess with intestinal haemorrhage. Br Med J.

